# Parasitic Myoma: A Rare Complication of Laparoscopic Myomectomy

**DOI:** 10.7759/cureus.78230

**Published:** 2025-01-30

**Authors:** Hung S Ho, Minh N Ho, Quyen H Dinh, Son T Trinh

**Affiliations:** 1 Obstetrics and Gynecology, National Hospital of Obstestrics and Gynecology, Hanoi, VNM; 2 Physiology and Human Biology, Hanoi University of Science, Vietnam National University, Hanoi, VNM; 3 Obstetrics and Gynecology, National Hospital of Obstetrics and Gynecology, Hanoi, VNM; 4 Obstetrics and Gynecology, Military Institute of Clinical Embryology and Histology, Vietnam Military Medical University, Hanoi, VNM

**Keywords:** gynecology, in-bag morcellation, laparoscopy, parasitic myoma, surgery

## Abstract

The occurrence of a parasitic myoma is an unusual condition and it is traditionally thought to be a variant of pedunculated subserosal myoma that has become separated from the uterus and received alternative blood supply. However, the prevalence of laparoscopic surgery might have brought a new type of parasitic myoma named iatrogenic parasitic myoma. This type of parasitic myoma raises the question of whether it should be considered as a long-term complication of laparoscopic myomectomy. This report aims to present a case of parasitic myoma in a 38-year-old woman who underwent laparoscopic myomectomy using a morcellator. Due to the prevalence of laparoscopic myomectomies and the risk of spreading unsuspected leiomyosarcoma with morcellation, this case highlights the need for in-bag containment morcellation to extract specimens. At the same time, a careful inspection and washing of the abdominopelvic cavity at the end of the surgery should be performed to minimize the complication of open power morcellation.

## Introduction

Uterine leiomyomas are benign smooth tumors of the uterus and occur in approximately 25% of women of reproductive age [1]. Surgery is considered for symptomatic patients for the treatment of leiomyomas. In recent years, advances in surgical equipment and techniques have made laparoscopy the surgical treatment of choice. Morcellation is a technique used to extract large masses through small incisions in minimally invasive surgery [2,3]. Although this allows for minimally invasive procedures with faster recovery, this still carries risks, such as spreading small specimens, requiring careful precautions like specimen retrieval bags.

Kelly and Cullen first introduced parasitic myomas in the 1900s [4]. Traditionally, it was thought to be the rare variant of pedunculated subserosal myoma that has become partially or completely separated from the uterus and received an alternative blood supply from other sources, such as the omentum and mesenteric vessels [5]. 

Parasitic myomas are rare; however, there has been an increase in parasitic myomas after laparoscopic myomectomy with the use of a morcellator. The overall incidence of parasitic fibroids following laparoscopic surgery involving morcellation has been reported to range from 0.12% to 0.9% [6-8]. The prevalence of laparoscopic surgery might have induced a new type of parasitic myoma named iatrogenic parasitic myoma. This type of parasitic myoma raises the question of whether it should be considered a long-term complication of laparoscopic myomectomy. This report reviews a case of a parasitic myoma in a patient who underwent laparoscopic myomectomy using a morcellator.

## Case presentation

A 38-year-old Vietnamese woman was admitted to our hospital with a complaint of progressively increasing abdominal distension. She indicated that she had undergone uterine myomectomy laparoscopy with power morcellation three years ago.

**Figure 1:**
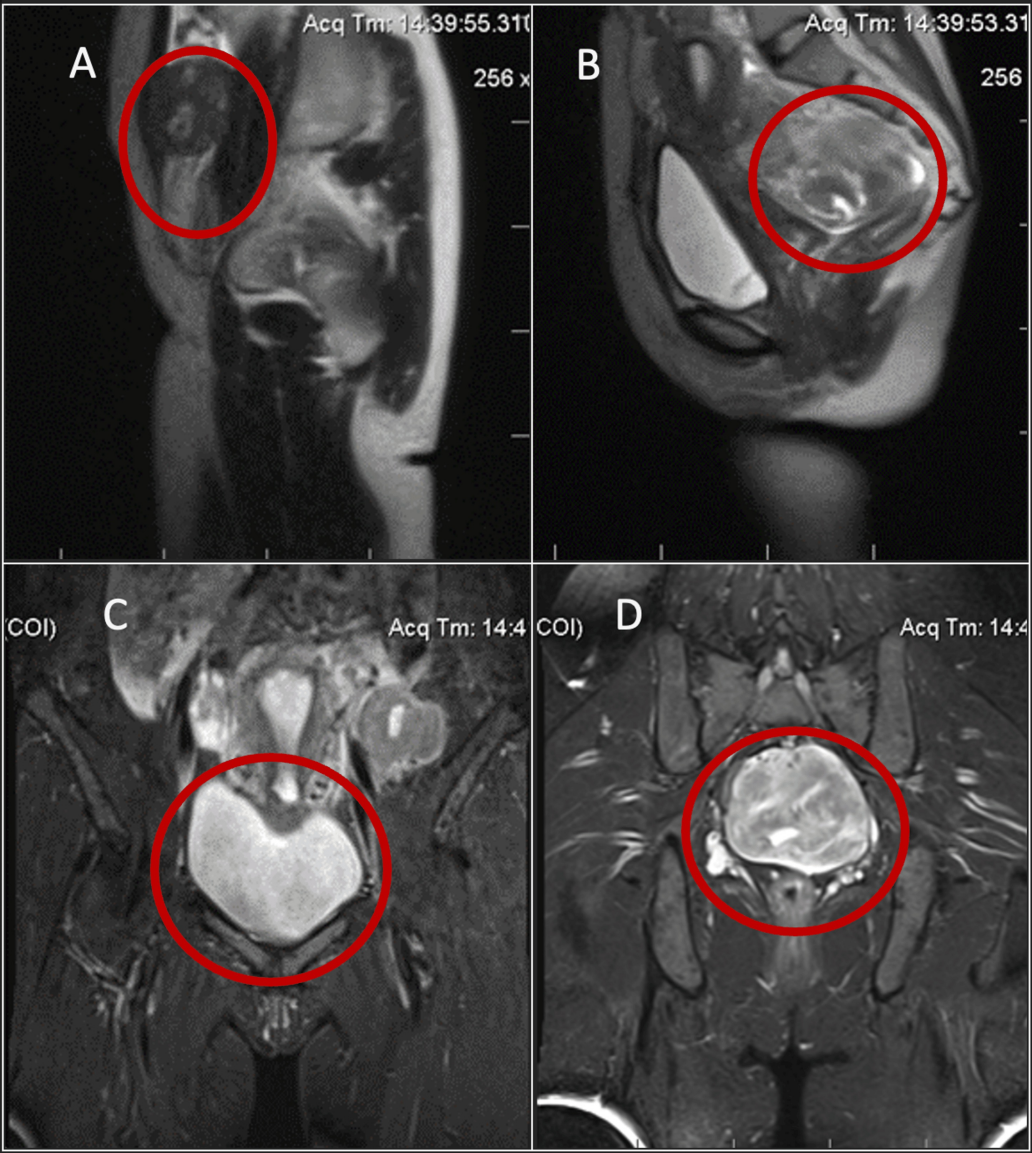
A mass with signal intensities that is similar to the myoma located in the abdominal wall was detected (A). An abnormal mass was located in the posterior wall of the uterus, measuring 90x68x95mm. The mass had well-defined margins and clear boundaries, with a signal intensity similar to the uterine myometrium, causing compression of the rectum (B). After contrast administration, the mass demonstrated strong and heterogeneous enhancement (C). Below the left ovary, a similar mass measuring 52x43x54mm was detected (D).

Gynecology examination showed the size of the uterus increased as large as that of 12 weeks of pregnancy. On MRI, masses with signal intensity similar to that of skeletal muscle and smooth muscle on both the T1 weighted image (T1WI) and T2 weighted image (T2WI) were noted (Figure 1).

**Figure 2:**
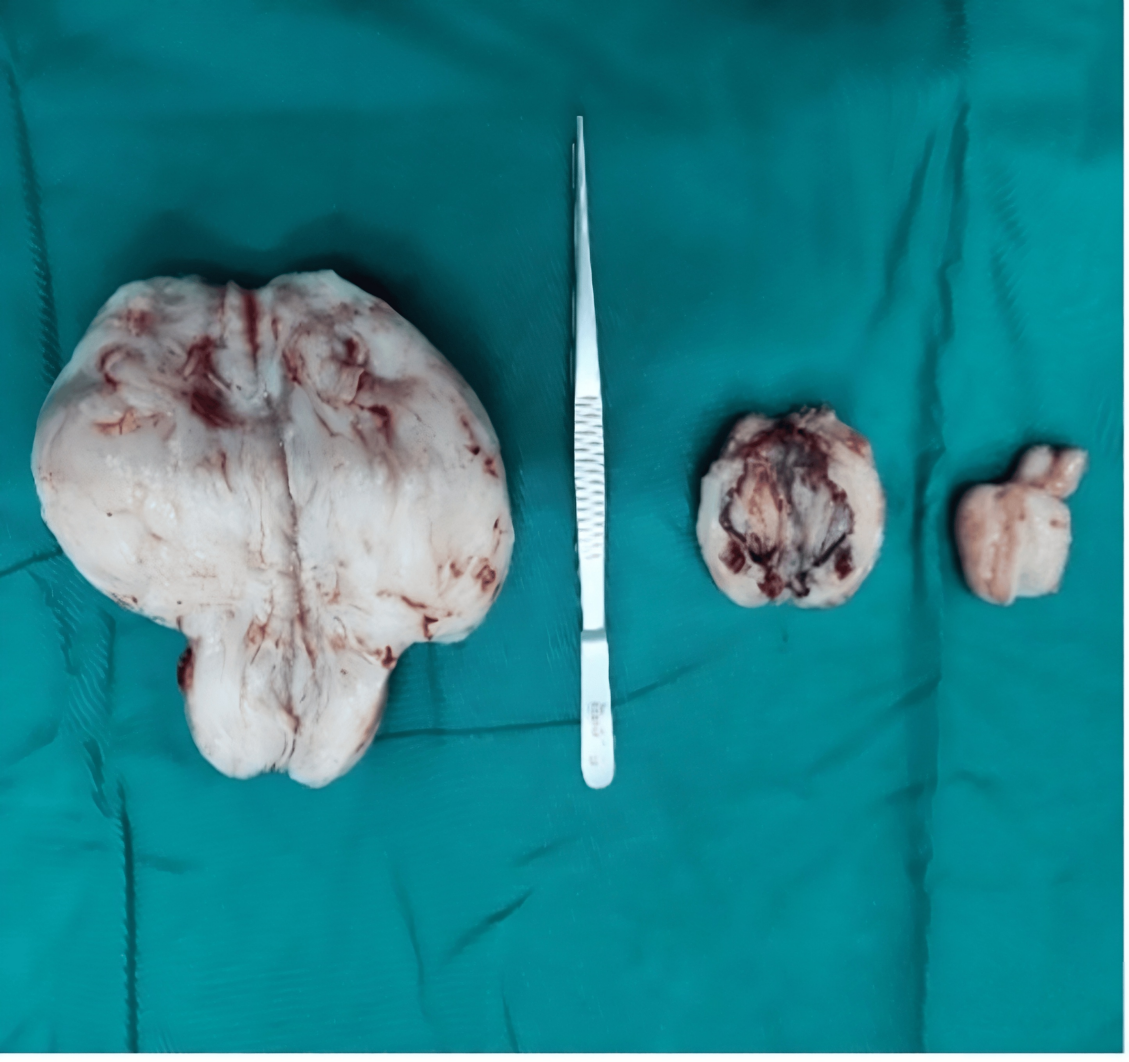
The postoperative tumor specimen included three fragments with a total weight of 400 grams.

**Figure 3:**
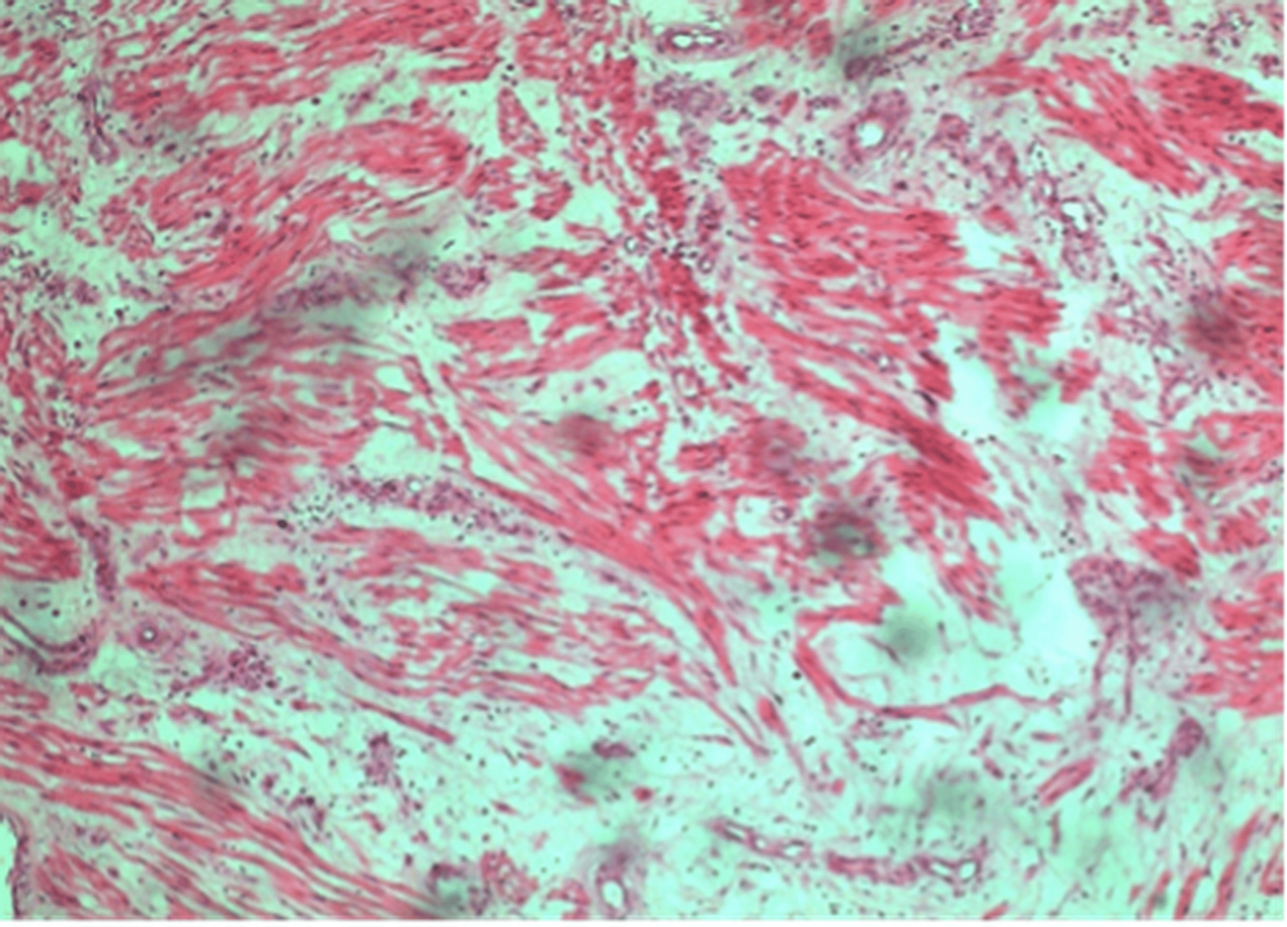
The tumor fragments consisted of spindle-shaped cells arranged in interwoven fascicles. The nuclei were small, round, and uniform, with no evidence of mitosis or necrosis. Areas of cystic degeneration and eosinophilic degeneration were observed. No atypical cells were detected.

The patient underwent exploratory laparotomy. During the surgery, we found that a large mass with a diameter of 10 cm originated from the retroperitoneum and adhered to the posterior wall of the uterus. At the same time, there were two smaller masses, approximately 2-4 cm, attached to the left parietal peritoneum near the uterus (Figure 2). In addition, a normal uterus, bilateral tubes, and ovaries were visualized. We resected all three masses without injuring the intestines, vessels, ureters, and bladder. The patient had an unremarkable postoperative course and reported asymptomatic to date. Figure 3 shows the structure of the tumor consisting of spindle-shaped cells with small, round nuclei, which is similar to the myoma's structure. 

## Discussion

Uterine leiomyomas are the most common benign tumors in premenopausal women [9, 10], and the diagnosis is straightforward in most cases. However, the diagnosis of a parasitic myoma is difficult because the symptoms are nonspecific. Patients often presented with abdominal distension or pelvic pain. A parasitic myoma can occur at various sites and vary in diameter, and sometimes the symptoms reflect its complication. Cho et al. reported a case of torsion of a parasitic mass with a twisted omental pedicle [11]. Fulcher and Szucs revealed a study of leiomyomatosis peritoneal dissemination complicated by ovarian torsion [12]. In both cases, patients presented with acute abdominal pain. A parasitic myoma can rarely mimic gastroenterological diseases with chronic constipation only [10].

Due to vague symptoms, an investigation should be conducted to assess abdominal mass properly. Sonographic findings in our case and that of others reported include solid and complex soft tissue masses that mimic a leiomyomatous uterus [12-15]. Our diagnosis was only confirmed by surgery and histopathology. However, MRI and CT help investigate myoma and find out its complications.

Parasitic myomas should be differentiated from leiomyomatosis peritonealis disseminata (LPD) (Table 1) [4, 16-18]. While a parasitic myoma is often large and solitary, LPD is characterized by multiple subperitoneal or peritoneal nodules (generally <1 cm in diameter) disseminated through the omentum and peritoneum. In LPD, there are countless numbers of nodules, which often mimic peritoneal carcinomatosis, but LPD often has a favorable prognosis [19].

**Table 1:** Parasitic myoma vs. leiomyomatosis peritonealis disseminata (LPD) Adapted from [4, 16-18].

Criteria	Parasitic myoma	Leiomyomatosis peritonealis disseminata (LPD)
Origin	Detached fibroid reattaching and growing	Multiple small smooth muscle nodules in the peritoneum
Relation to surgery	Yes, often after morcellation	No, spontaneous
Cause	Iatrogenic (due to surgery)	Hormonal influence (pregnancy, hormone therapy)
Imaging features	Single well-defined mass	Multiple nodules disseminated
Histology	Similar to the original uterine fibroid	Benign, atypical smooth muscle nodules

The pathogenesis of parasitic myoma can be divided into two broad categories. Parasitic myomas spontaneously develop from pedunculated myomas and iatrogenic myomas associated with the restriction of blood supplies to the uterus or previous uterine surgery [20].

Kelly and Cullen described spontaneous parasitic myomas as originating from pedunculated myomas that are still partially attached to the uterus but somehow gained their main blood supply from other organs and became detached and parasitic to the omentum and mesentery [4]. In contrast to spontaneous parasitic myomas, in theory, iatrogenic myomas can be a result of any process that restricts the blood supply to the uterus. Ceana et al. reported two cases of parasitic myoma formation, one free from the uterus after administration of leuprolide acetate and one after magnetic resonance imaging guided-focused ultrasound (MRgFUS) treatment of uterine myomas [20]. However, in our case, the patient had a history of laparoscopic myomectomy using a power morcellator, and she came back to us after 36 months for parasitic myoma. Such post-laparoscopic myomectomy myomas have been reported in the literature. This frequency leads us to think pieces of myoma missed at the morcellation due to incomplete removal could grow intraperitoneally and cause parasitic myoma. Therefore, a new iatrogenic mechanism of parasitic myomas has been associated with laparoscopic surgery with the use of a morcellator. Although the advantages of laparoscopic surgery compared to laparotomy are fully proven, such as lesser blood loss, less pain, lower morbidity, and minimal hospital stay, removal of an excised uterine myoma is rather challenging in many cases [21]. Large tissues such as the uterus and leiomyomas are often fragmented by power morcellators to aid in tissue removal. Some of the resultant small fragments may inadvertently remain in the abdominal cavity and become implanted with the development of blood supply, resulting in subsequent growth. Van der Meulen et al. estimated the incidence of iatrogenic parasitic myoma to be 0.12% to 0.94% [22].

Management of parasitic myomas has been reported mostly by excision. In our case, all the parasitic myomas were excised without any complication. However, the excision of myoma can be difficult if it involves the bowel and uterus, and the patient should be informed about the extensive surgery. A parasitic myoma firmly attached to peritoneal tissue adjacent to the ureter has been reported in the review by Takeda et al. and thus a ureteral stent had to be placed in the left ureter before dissection [23]. Laibangyang et al. presented a case of a parasitic myoma causing bowel perforation, and small bowel anastomosis had to be performed [24].

Owing to an increase in parasitic myomas after laparoscopic myomectomy, they should be recognized as a long-term complication of power morcellation in laparoscopic myomectomy. In addition, power morcellation is associated with an increased risk of spreading unsuspected cancerous tissues in women undergoing myomectomy for presumed benign leiomyomas, leading to a worsened prognosis [25]. Therefore, even though the incidence of parasitic myomas is low, clinicians should be aware of it, and this condition should be explained to patients and families before surgery as an unusual late complication of myoma tissue morcellation. This complication can be prevented by performing a colpotomy or mini-laparotomy to retrieve intact myomas. Another alternative way is in-bag containment morcellation, which was proven to be an efficient technique to reduce the risk of intraperitoneal dispersion of tissue fragments [26].

## Conclusions

Laparoscopic myomectomy is a commonly performed procedure in gynecology, aimed at removing uterine fibroids while minimizing invasiveness. Several techniques are available for extracting fibroids from the abdominal cavity. Among these, morcellation offers the advantage of extracting specimens through a small trocar incision, reducing the need for larger incisions and maintaining the benefits of a minimally invasive approach. However, this technique risks specimen dissemination and potential implantation of tumor fragments within the peritoneal cavity, which may lead to regrowth in two to three years after surgery. In many cases, laparotomy is preferred to ensure complete removal of the tumor, particularly when dissemination has affected multiple sites. To minimize the risk of recurrence, we recommend performing in-bag morcellation to prevent the spread of small tumor fragments, combined with thorough irrigation of the abdominal cavity. In addition, monitoring patients after surgery is crucial in detecting complications early and ensuring recovery aligns with expectations. Regular follow-ups enable timely intervention, especially in cases with potential risks like tissue dissemination or recurrence. Updating guidelines based on emerging evidence, such as the use of in-bag morcellation, can further enhance patient safety and minimize complications. 
